# Synolitic Graph Neural Networks for MRI-Derived Radiomic-Based Prediction of Prostate Cancer Progression on Active Surveillance

**DOI:** 10.3390/cancers18091389

**Published:** 2026-04-28

**Authors:** Mikhail I. Krivonosov, Arseniy Trukhanov, Nikita Sushentsev, Tristan Barrett, Alexey Zaikin

**Affiliations:** 1 Research Center in Artificial Intelligence, Institute of Information Technologies, Mathematics and Mechanics, Lobachevsky State University, Nizhny Novgorod 603022, Russia; 2 Mriya Life Institute, National Academy of Active Longevity, Moscow 124489, Russia; 3 Department of Radiology, University of Cambridge School of Clinical Medicine, Cambridge CB2 0QQ, UK; 4 Institute for Cognitive Neuroscience, University Higher School of Economics, Moscow 101000, Russia; 5 Department of Mathematics and Women’s Cancer, University College London, London WC1E 6BT, UK

**Keywords:** prostate cancer, active surveillance, progression prediction, radiomics, graph neural networks, Synolitic Graph Neural Networks, machine learning, MRI

## Abstract

**Background:** Prostate cancer (PCa) is one of the most prevalent malignancies in men, and active surveillance (AS) is the recommended management strategy for low- and favourable intermediate-risk disease. Predicting which patients will progress during AS remains a clinical challenge. MRI-derived radiomics has shown promise for risk stratification, but conventional machine learning approaches treat radiomic features as independent variables and may not capture the complex inter-feature dependencies within imaging data. This study evaluates the application of Synolitic Graph Neural Networks (SGNNs) to baseline MRI-derived radiomic features for predicting prostate cancer progression on active surveillance. **Methods:** We studied 343 AS patients (73 progressors, 270 non-progressors) from a single-centre cohort prospectively enrolled between 2013 and 2019 and retrospectively analysed. Seventy-two radiomic features were extracted from baseline 3T MRI (T2-weighted imaging and apparent diffusion coefficient maps), together with three clinical variables (prostate volume, PSA, PSA density). The SGNN pipeline transformed each patient’s feature profile into a weighted graph encoding pairwise feature relationships via logistic regression classifiers trained within each cross-validation fold. GCN and GATv2 architectures were evaluated with multiple sparsification strategies and compared against Gradient Boosting, SVM, Random Forest, and logistic regression using 5-fold stratified cross-validation. **Results:** Among conventional methods, Gradient Boosting achieved the highest ROC-AUC (0.634 ± 0.080). The SGNN pipeline with GATv2, confidence-based sparsification (*p* = 0.8), and extended node features incorporating graph centrality measures achieved the best performance (ROC-AUC = 0.699 ± 0.044), an absolute improvement of 0.065 over the best conventional method. The addition of topological node features consistently improved performance by 3–5% across configurations. GATv2 outperformed GCN in matched settings. **Conclusions:** As a proof of concept, the SGNN framework achieved the highest mean ROC-AUC among the evaluated single-timepoint approaches, though results require confirmation in independent external cohorts. By encoding inter-feature relationships as patient-specific graphs, SGNN offers a complementary modelling paradigm for radiomic data in clinical oncology. Future work should incorporate longitudinal data and graph explainability methods.

## Simple Summary

Prostate cancer patients on active surveillance require accurate risk stratification to identify those likely to progress. This study evaluated whether Synolitic Graph Neural Networks (SGNNs) applied to baseline MRI radiomic features could improve prediction of progression compared with conventional machine learning methods. Data from 343 patients (73 progressors, 270 non-progressors) were analysed using 72 radiomic features from T2-weighted MRI and ADC maps, together with prostate volume, PSA, and PSA density. The SGNN approach represented each patient as a graph capturing relationships between features and was compared with Gradient Boosting, Random Forest, SVM, and logistic regression using cross-validation. The best conventional model achieved an ROC-AUC of 0.634, whereas the SGNN model using a GATv2 architecture reached an ROC-AUC of 0.699. Incorporating graph-based feature relationships improved performance by 3–5%. These results suggest SGNN provides a promising alternative framework for radiomic-based prediction in prostate cancer active surveillance.

## 1. Introduction

Prostate cancer (PCa) is the second most frequently diagnosed malignancy and the fifth leading cause of cancer-related mortality among men worldwide, with its incidence expected to continue rising due to an ageing male population [1,2]. In the UK, approximately 43% of men present with low- or favourable intermediate-risk localised disease [3], for which level 1 evidence supports the non-inferiority of active surveillance (AS) to radical treatment in terms of 10-year survival [4,5,6,7]. AS permits the deferral of radical treatment in the absence of disease progression, thereby reducing treatment-related morbidity without compromising oncological outcomes [8].

Long-term registry data confirm that AS is safe, with 49% of patients remaining progression-free at 10 years and prostate cancer-specific mortality of only 0.1% [9]. However, histopathological upgrading occurs in 22–38% of men during AS [10,11], and a cumulative five-year AS discontinuation rate of 44% has been reported, with 27% triggered by disease progression [12,13]. These figures underscore the unmet clinical need for robust baseline risk-stratification tools that can identify patients harbouring lesions with high progressive potential, either for intensified monitoring or early treatment escalation [14].

Magnetic resonance imaging (MRI) has become integral to AS programmes, primarily owing to its high negative predictive value for clinically significant disease [15,16]. Several studies have confirmed that the presence of an MR-visible lesion at baseline is associated with a higher risk of progression [14,17,18]. The Prostate Cancer Radiological Estimation of Change in Sequential Evaluation (PRECISE) scoring system was introduced to standardise longitudinal MRI reporting during AS [19], yet serial MRI retains a limited positive predictive value and cannot replace repeat biopsy [20,21]. No systematic attempts have been made to further stratify MR-visible lesions based on their progressive potential using quantitative imaging biomarkers. The inherent difficulty of baseline risk stratification lies in the ‘spectrum effect.’ Because patients are strictly pre-selected for inclusion in AS based on low- or favourable intermediate-risk criteria, the resulting cohort is relatively homogeneous. In this context, the imaging ‘signals’ that differentiate a future progressor from a non-progressor are exceedingly subtle at the point of enrolment, often predating visible radiological changes.

These limitations have motivated the development of radiomics—the high-throughput extraction of quantitative intensity, shape, and texture features from medical images that are imperceptible to visual assessment [22,23]. In prostate cancer, radiomics has been applied to improve detection of clinically significant disease [24,25,26] and to predict histopathological upgrading from biopsy to radical prostatectomy [27]. In a proof-of-concept study on the same institutional AS cohort examined here, Sushentsev et al. [28] demonstrated that combining T2WI-derived radiomic features with PSA and PSA density improved the AUC for predicting progression from 0.61 to 0.75 using leave-one-out cross-validation on 73 lesions. Subsequent longitudinal extensions of this work achieved AUCs of 0.91 with delta-radiomic models [29] and 0.92 with time-series approaches [30], demonstrating that temporal information substantially enhances progression prediction. More recently, Vershinina et al. [31] applied explainable artificial intelligence methods to the same cohort, reporting an AUC of 0.793 with SHAP-based feature selection.

While these studies have demonstrated the value of radiomics for AS monitoring, all employed conventional machine learning methods that modelled radiomic features as independent, unstructured variables. Graph Neural Networks (GNNs) are deep learning models designed to learn from relational, graph-structured data through message-passing operations [32,33]. The Synolitic Graph Neural Network (SGNN) framework—synolitic denoting the collective encoding of pairwise relationships into a unified graph representation—recently introduced by Krivonosov et al. [34] provides a principled method for converting high-dimensional tabular data into patient-specific graph representations, in which nodes correspond to features, and edge weights encode the discriminative relationships between feature pairs learned from lightweight pairwise classifiers. This approach has been applied to cancer biomarker discovery, including pancreatic cancer proteomic profiling [35,36], where it demonstrated the ability to capture network-level dysregulation patterns.

In this study, we apply the SGNN framework to predict prostate cancer progression on active surveillance using baseline MRI-derived radiomic features in a cohort of 343 AS patients. We evaluate two GNN architectures (GCN and GATv2) with multiple sparsification strategies and compare their performance against conventional machine learning benchmarks. To our knowledge, this is the first application of graph neural networks to radiomic data for predicting cancer progression in an active surveillance setting.

## 2. Materials and Methods

### 2.1. Study Cohort

This retrospective analysis used data from 343 consecutive patients with biopsy-confirmed PCa enrolled on the AS programme at Cambridge University Hospitals NHS Foundation Trust between July 2013 and October 2019. The study was approved by the local institutional review board (NRES Committee East of England—Cambridge South, UK) as part of a service evaluation of the prostate diagnostic pathway, with the requirement for individual informed consent waived. All procedures were conducted in accordance with the Declaration of Helsinki.

Patients were enrolled on AS according to previously published local eligibility criteria [37] and were required to have at least one MR-visible lesion, a minimum two-year follow-up, and at least two consecutive 3T MRI examinations performed on the same scanner. Exclusion criteria comprised the absence of an MR-visible lesion, prior or interim treatment for PCa or benign prostatic disease, and the presence of pelvic metallic implants.

The cohort comprised 73 progressors (21.3%) and 270 non-progressors (78.7%). Disease progression was defined as histopathological upgrading in the ISUP grade group on repeat targeted biopsy, prompting transition to radical treatment. The non-progressor group consisted of patients with stable disease throughout follow-up, defined as no radiological progression (PRECISE score 3 [19]) and no histopathological upgrading on all repeat targeted biopsies. Baseline clinicopathological characteristics are summarised in Table 1.

The shorter median AS duration in progressors (40 months) compared with non-progressors (49 months) reflects the study design: progressors exited the AS programme at the point of histopathological upgrading, while non-progressors were followed until the study cut-off. All patients, regardless of outcome, were required to have a minimum two-year follow-up before inclusion. This design means the binary class labels reflect outcome status at last follow-up; patients classified as non-progressors may include a small proportion who would have progressed with longer observation—a form of informative censoring inherent to retrospective binary classification in AS cohorts.

### 2.2. MRI Acquisition and Radiomic Feature Extraction

All patients underwent prostate MRI on a 3T MR750 scanner (GE Healthcare, Waukesha, WI, USA) using a 32-channel receiver coil. An intravenous injection of hyoscine butylbromide (Buscopan, 20 mg/mL; Boehringer, Ingelheim am Rhein, Germany) was administered prior to imaging to minimise bowel peristalsis, unless clinically contraindicated. The multiparametric protocol comprised multiplanar high-resolution T2-weighted 2D fast-recovery fast-spin-echo sequences (field of view 18 × 18 cm^2^; voxel size 0.35 × 0.35 mm^2^; slice thickness 3 mm; gap 0 mm) and diffusion-weighted imaging using a spin-echo echo-planar imaging pulse sequence (b-values: 150, 750, and 1400 s/mm^2^), from which apparent diffusion coefficient (ADC) maps were calculated automatically. Full acquisition details have been described previously [28].

Tumour regions of interest (ROIs) were delineated on T2-weighted images and ADC maps by a fellowship-trained uro-radiologist with 14 years’ experience in prostate MRI and an imaging research fellow with 5 years’ experience, working in consensus using the open-source ITK-SNAP segmentation software version 4.0 [38]. Segmentation reliability was evaluated through morphological perturbation using opening and closing operations with a 3D spherical structuring element of 1-pixel radius [28].

Radiomic features were extracted using PyRadiomics version 2.0 [39] in compliance with the Image Biomarker Standardization Initiative (IBSI) recommendations [40]. Six feature classes were computed: first-order intensity statistics, Grey-Level Co-occurrence Matrix (GLCM), Grey-Level Run-Length Matrix (GLRLM), Grey-Level Size Zone Matrix (GLSZM), Grey-Level Dependence Matrix (GLDM), and shape-based 3D features. Feature robustness was assessed using the intraclass correlation coefficient (ICC) following ROI perturbation, with a cut-off of ICC > 0.8 applied to retain only highly robust features. After this filtering, 72 radiomic features were retained for the present analysis. Three clinical variables were additionally included: baseline prostate gland volume, PSA, and PSA density (PSAd).

### 2.3. Conventional Machine Learning Benchmarks

Four conventional ML algorithms were evaluated: (1) Gradient Boosting (100 estimators, max depth 6, learning rate 0.1); (2) Support Vector Machine (SVM) with radial basis function kernel, automatic gamma scaling, and class weight balancing; (3) Random Forest (100 trees, max depth 10, class weight balancing); and (4) logistic regression with L-BFGS optimisation and class weight balancing. All features were standardised using z-score normalisation prior to training.

### 2.4. Synolitic Graph Neural Network Pipeline

The SGNN pipeline [34] converted each patient’s tabular radiomic profile into a weighted graph through several sequential steps. First, mutual-information-based feature selection identified the top N most informative features ($N\;\in \;\left\{20,\;30,\;40\right\}$). For each pair of selected features, a logistic regression classifier was trained using only those two features, yielding $N\left(N-1\right)/2$ pairwise models. Each pairwise classifier was implemented as a standard logistic regression (L-BFGS solver, maximum 1000 iterations, L2 regularisation with C = 1.0, no class weighting), trained on the two-feature subspace within the current training fold. The predicted probability P(y = 1|fi, fj) was then evaluated for each patient in the held-out test fold to construct that patient’s edge weight, ensuring no information leakage from test to training data. Both feature selection and pairwise classifier training were performed strictly within the current cross-validation training fold; test fold samples were never used for graph construction, thereby preventing information leakage.

For each patient, a graph $G\;=\;\left(V,\;E\right)\;$ was constructed where nodes V corresponded to the selected features and edge weights $w_{ij}\;=\;2\;\cdot \;|P(y=1|f_{i},\;f_{j})\;-\;0.5|\;$ encoded the pairwise discriminative confidence for that specific patient. This created a patient-specific weighted adjacency matrix reflecting how discriminative each feature pair was for that individual.

For each node, five features were computed: the original standardised feature value, normalised degree centrality, weighted strength (sum of incident edge weights), closeness centrality (inverse of mean shortest path distance to all other nodes), and betweenness centrality (fraction of shortest paths passing through the node). These topological descriptors encoded each feature’s structural role within the patient-specific graph.

### 2.5. Graph Sparsification

Three sparsification strategies were evaluated: (1) no sparsification, using the full weighted graph; (2) confidence-based sparsification, retaining the top p fraction of edges ranked by classifier confidence $\left|w_{ij}\;-\;0.5\right|$, with $p\;\in \;\left\{0.2,\;0.8\right\}$ tested; and (3) minimum connected sparsification, using binary search to find the maximum threshold that maintained graph connectivity, producing the sparsest connected graph.

### 2.6. GNN Architectures and Hyperparameter Optimisation

Two GNN architectures were evaluated. Graph Convolutional Networks (GCNs) [41] aggregate neighbour features through spectral convolutional filters. Graph Attention Networks v2 (GATv2) [42] employ a dynamic self-attention mechanism that learns the importance of different neighbours for each node. Both architectures were implemented with 3 layers, edge encoder MLPs to incorporate edge weights, residual connections, batch normalisation, dropout, and global mean and max pooling for graph-level representation, followed by a two-layer classification head. Models were trained using the Adam optimiser with class-weighted cross-entropy loss to address class imbalance.

Hyperparameters were tuned using Optuna Bayesian optimisation [43] with 8 trials per configuration. The search space comprised hidden dimensions ∈ {32, 64, 128}, dropout rate in [0.0, 0.5], and learning rate in [10^−4^, 10^−2^]. Optuna selected the best hyperparameters based on validation performance within the training fold of each cross-validation iteration, ensuring that the held-out test fold was not used for model selection.

### 2.7. Simplified SGNN and Evaluation Protocol

A simplified SGNN approach was also evaluated, in which graph-derived topological features (centrality measures, global graph properties, edge weight statistics) were extracted from each patient’s synolitic graph and used as input to conventional Gradient Boosting (SGNN-GB) and Random Forest (SGNN-RF) classifiers, bypassing end-to-end GNN training.

All models were evaluated using 5-fold stratified cross-validation, chosen over a single train/test split to provide more robust performance estimates given the moderate sample size and class imbalance. The primary metric was the area under the receiver operating characteristic curve (ROC-AUC), with F1-score and accuracy reported as secondary metrics. Results are presented as mean ± standard deviation across the five folds. Formal 95% confidence intervals are not reported, as with five-fold-level observations the t-distribution estimate (4 degrees of freedom) produces intervals too wide to be meaningfully interpreted; the standard deviation across folds is the more honest summary of fold-to-fold variability in this setting.

## 3. Results

### 3.1. Cohort Characteristics

Baseline cohort characteristics are summarised in Table 1. PSA density was significantly higher in progressors than in non-progressors (median 0.14 vs. 0.12; *p* = 0.014), consistent with previous reports from this cohort [28]. Baseline PSA and gland volume did not differ significantly between groups. Among progressors, the median time on AS before progression was 40 months, whereas among non-progressors, the median follow-up was 49 months (*p* = 0.001).

### 3.2. Conventional Machine Learning Performance

**Figure 1:**
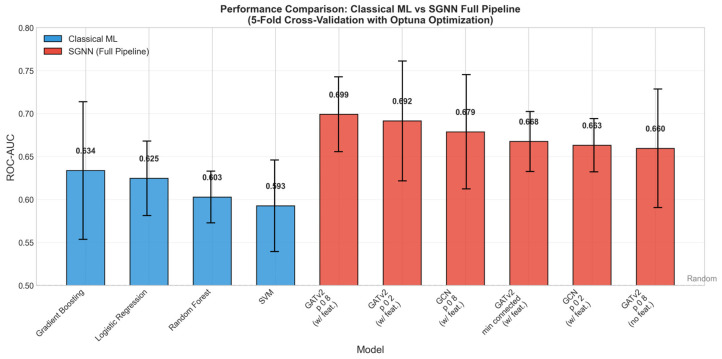
Comparison of all evaluated methods. Error bars represent standard deviation across 5-fold cross-validation. GATv2 with confidence-based sparsification (*p* = 0.8) and extended node features achieved the highest mean ROC-AUC (0.699).

**Figure 2:**
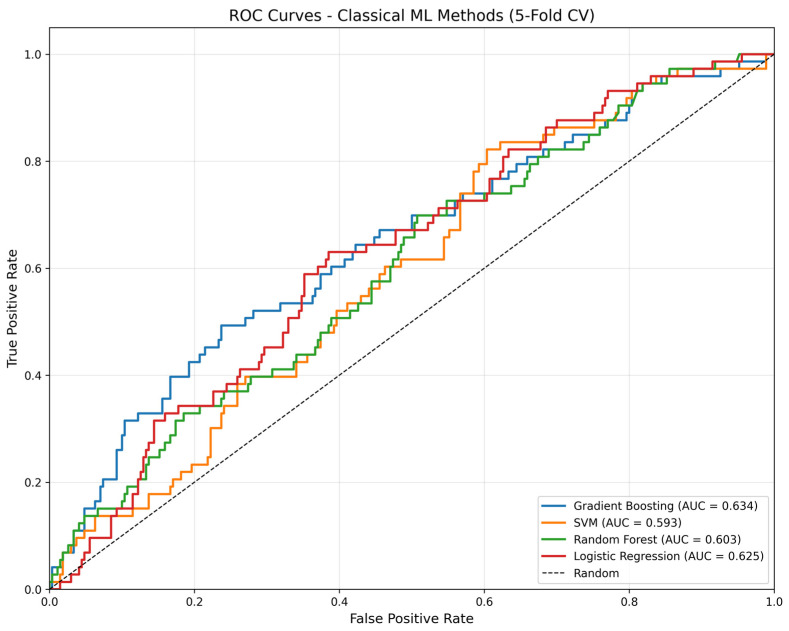
ROC curves for conventional machine learning methods aggregated across all folds. Gradient Boosting (AUC = 0.634) and logistic regression (AUC = 0.625) showed the best discriminative performance among conventional approaches.

Figure 1 provides a comprehensive visual comparison of all evaluated methods. Table 2 presents conventional ML performance, with corresponding ROC curves shown in Figure 2. Gradient Boosting achieved the highest mean ROC-AUC (0.634 ± 0.080), though with considerable variance across folds. Logistic regression demonstrated more stable performance (0.625 ± 0.043) and the best F1-score (0.365). The uniformly low F1-scores across all methods reflect the difficulty of correctly identifying the minority class in this imbalanced dataset (21% progressors).

### 3.3. SGNN with Full GNN Pipeline

Table 3 presents the full SGNN pipeline results across architectures, sparsification strategies, and node feature configurations. The best-performing configuration was GATv2 with confidence-based sparsification (*p* = 0.8) and extended node features, achieving a mean ROC-AUC of 0.699 ± 0.044—an absolute improvement of 0.065 over the best conventional method.

**Figure 3:**
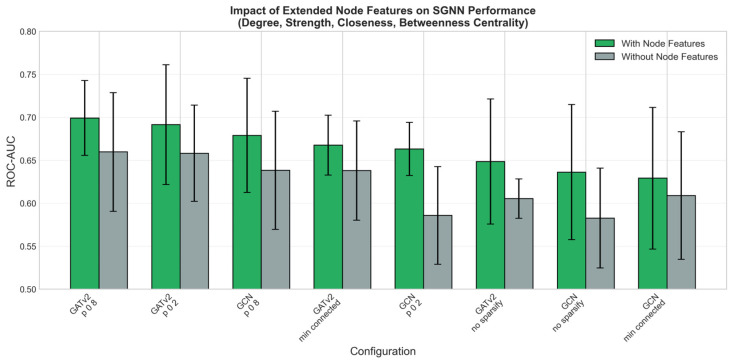
Impact of extended node features on SGNN performance. Configurations incorporating graph centrality features consistently outperformed scalar-only configurations by 3–5% in ROC-AUC across all sparsification methods.

**Figure 4:**
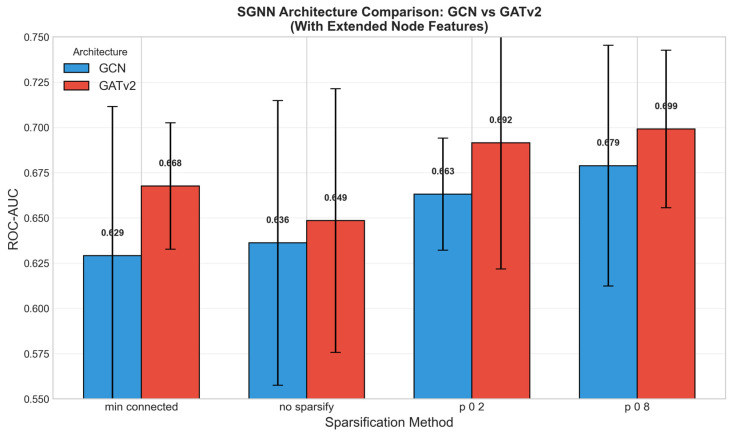
Comparison of GCN and GATv2 architectures across sparsification methods with extended node features. GATv2 outperformed GCN in all matched configurations.

Three consistent patterns emerged from the systematic evaluation. First, incorporating graph centrality features (degree, strength, closeness, betweenness) added 3–5% to the ROC-AUC across all configurations (Figure 3), indicating that the topological role of each radiomic feature within the patient-specific network carries predictive information beyond its raw value. Second, GATv2 outperformed GCN in all matched configurations (Figure 4), suggesting that the dynamic attention mechanism is better suited for learning discriminative patterns from radiomic feature graphs. Third, confidence-based sparsification with *p* = 0.8 (retaining more high-confidence edges) consistently outperformed both the fully connected graph and the minimum connected approach, indicating that moderate edge pruning optimally balances signal preservation and noise removal.

To assess whether the SGNN improvement was driven by the clinical variables (PSA, PSA density, gland volume) or by the radiomic inter-feature graph structure, we examined the retention frequency and mutual information rank of these variables across the five CV folds for the best-performing configuration (GATv2, confidence-based sparsification *p* = 0.8, top 30 of the full 75-feature pool). PSA density was retained in three of five folds but with highly variable rankings (best rank three, worst rank 75); PSA showed a similar pattern (three of five folds; best rank 19, worst rank 70); gland volume was not retained in any fold. The extreme rank variability of PSA density—ranging from the third most informative feature in one fold to the least informative in another—reflects the instability of mutual information estimation in small, imbalanced training sets rather than a consistent clinical signal. The inconsistent retention of clinical variables across folds indicates that the SGNN’s performance advantage over conventional methods cannot be attributed to systematic preferential selection of PSA density or PSA. The radiomic inter-feature graph structure contributes to the predictive value independently of clinical variables, as evidenced by consistent SGNN outperformance even in folds where neither PSA density nor PSA was selected. Future work should evaluate whether treating PSA density as a mandatory model input—rather than a data-driven selection candidate—further improves performance.

### 3.4. Simplified SGNN and Overall Comparison

The simplified SGNN approach, which extracts graph-derived topological features for use with conventional classifiers, achieved a maximum ROC-AUC of 0.579 ± 0.079 (SGNN-GB with 40 features; Table 4). This was lower than conventional Gradient Boosting applied directly to raw features (0.634), indicating that the observed improvement from the full SGNN pipeline stems from end-to-end GNN learning over the graph structure rather than from graph-derived summary statistics alone.

Figure 1 provides a comprehensive visual comparison of all evaluated methods.

## 4. Discussion

This study applied the Synolitic Graph Neural Network framework to predict prostate cancer progression on active surveillance using baseline MRI-derived radiomic features. The best SGNN configuration—GATv2 with confidence-based sparsification (*p* = 0.8) and extended node features—achieved a mean ROC-AUC of 0.699 ± 0.044, compared to 0.634 ± 0.080 for the best conventional method (Gradient Boosting), an absolute improvement of 0.065. Although no formal statistical comparison was performed—and with only five-fold-level AUC observations, such a test would be underpowered—the consistent direction of improvement across the large majority of SGNN configurations, 11 of 12, suggests a potentially robust signal that requires confirmation in independent data.

The consistent benefit of graph centrality features (3–5% improvement across all configurations) supports the hypothesis that the structural position of a radiomic feature within a patient’s inter-feature network carries predictive information beyond its raw value. This finding is analogous to observations in proteomic cancer biomarker studies, where the SGNN framework captured pathway-level coordination invisible to conventional models [35,36]. The superiority of GATv2 over GCN aligns with results from those proteomic applications [35], likely because the attention mechanism dynamically adjusts the influence of different feature-pair relationships on a per-patient basis [42]. It is important to note that stronger discrimination is expected—and has been demonstrated in this cohort—once longitudinal imaging data are incorporated [29,30]. The baseline single-timepoint setting imposes an inherent ceiling on discriminative performance due to the biological homogeneity of the pre-selected AS population. An ROC-AUC near 0.70 at this stage is therefore not a failure of the method but rather a reflection of the clinical reality that disease trajectory has not yet manifested in imaging phenotype at enrolment.

The biological interpretation of SGNN graph structures deserves elaboration. Edge weights encode the pairwise discriminative utility of radiomic feature pairs for a given patient, not direct biological quantities. However, features that emerge as high-weight edges across many patients—particularly cross-modality pairs coupling ADC-derived diffusion features with T2WI texture or shape descriptors—are biologically plausible candidates for imaging correlates of early tumour aggressiveness. The centrality measures assigned to each node identify radiomic features that occupy ‘hub’ positions in the inter-feature network, which may correspond to imaging phenotypes most correlated with the molecular or histological drivers of progression. Graph explainability analysis (e.g., GNNExplainer [44]) applied to the trained model would allow systematic identification of the most predictive feature interactions, providing both biological insight and a basis for prospective biomarker validation. This represents an important priority for future work.

These results should be contextualised within the broader literature on this institutional AS cohort. Sushentsev et al. [28] reported an AUC of 0.75 (95% CI 0.64–0.86) using a combined clinical and T2WI-derived radiomic model with KNN classification and leave-one-out cross-validation on 73 lesions. Several methodological differences make direct comparison with our results difficult: different sample sizes (73 vs. 343 patients), different cross-validation protocols (LOOCV vs. 5-fold CV), and different feature sets (their best model explicitly included PSA density as a clinical predictor alongside radiomic features). Subsequent longitudinal studies on this cohort achieved substantially higher performance—AUCs of 0.91 with delta-radiomic models [29] and 0.92 with time-series LSTM approaches [30]—underscoring that temporal information from serial scans substantially improves progression prediction beyond what any single-timepoint model can achieve.

A notable finding is that the simplified SGNN approach (graph features fed into Gradient Boosting) underperformed conventional Gradient Boosting applied directly to raw features (0.579 vs. 0.634). This demonstrates that the synolitic graph structure alone does not improve prediction when the downstream classifier is conventional; the performance gain arises specifically from end-to-end GNN learning, which exploits the full adjacency structure during message passing. This observation also provides evidence against overfitting of the full pipeline: a heavily overfit model would show inflated test performance, whereas the more parsimonious explanation for the SGNN advantage is that end-to-end message-passing exploits the adjacency structure in ways that graph summary statistics cannot capture. This has practical implications: the computational overhead of the full GNN pipeline is justified only when end-to-end training is employed.

From a clinical perspective, the appropriate utility threshold for a baseline risk stratification model in active surveillance must be framed in context. No model currently deployed in AS achieves an ROC-AUC above 0.80 from baseline imaging alone in a similarly pre-selected, biologically homogeneous cohort. The relevant comparator is not an idealised high-accuracy classifier but the clinical status quo, in which surveillance intensity is guided largely by PSA density and PI-RADS scores, tools that individually achieve AUCs in the range 0.60–0.65 for histopathological progression prediction in this cohort [28]. In this context, an absolute improvement of 0.065 in AUC, achieved without additional clinical variables, represents incremental value that could inform tiered surveillance pathways. The clinical role envisaged for such a model is not to replace biopsy or longitudinal MRI but to complement existing risk stratification by identifying, at baseline, patients who may benefit from earlier or more frequent re-evaluation. Whether this improvement translates to patient-level clinical benefit requires prospective validation, appropriate calibration, and decision-analytic modelling—steps that are beyond the scope of this proof-of-concept study but are important priorities for future work.

The 21.3% progression rate in this cohort is consistent with long-term AS registry data and reflects the clinical reality of a pre-selected low-risk population [9,45] rather than a sampling artefact. In the AS clinical context, the asymmetric consequences of under-detecting progression (delayed treatment escalation in a patient with aggressive disease) versus over-detecting it (unnecessary biopsy or early treatment in a patient who would remain stable) strongly favour operating at higher sensitivity thresholds. The reported ROC-AUC is a threshold-independent summary statistic and is therefore the most appropriate primary metric for comparing models in this imbalanced setting; the optimal operating point for clinical deployment would be determined by decision-analytic modelling of the specific sensitivity–specificity trade-off acceptable in the target clinical setting.

The moderate absolute performance of all single-timepoint models (AUCs in the range 0.59–0.70) reflects the inherent difficulty of predicting histopathological progression from a single baseline MRI. Cancer progression on AS is influenced by numerous biological and clinical factors that evolve over time, and a single snapshot may capture only a fraction of the relevant information. The substantial improvement achieved by longitudinal delta-radiomic [29] and time-series [30] approaches in prior work on this cohort underscores this point. However, a critical distinction must be made between the predictive power of a static snapshot and the diagnostic power of temporal trends. While longitudinal models benefit from observing the actual biological ‘velocity’ of the tumour over several years, the SGNN framework attempts to forecast this behaviour from a single time point. Given that the baseline AS population is ostensibly stable by definition, an ROC-AUC of 0.699 suggests that the synolitic graph structure captures early, non-linear feature interactions that are invisible to both conventional models and visual assessment at the time of enrolment. A natural extension of the SGNN framework would be dynamic graph construction incorporating longitudinal data, where edge weights evolve across serial scans to capture temporal changes in radiomic feature relationships.

We evaluated 12 SGNN configurations (two architectures × three sparsification strategies × two node feature settings) in addition to four conventional methods and six simplified SGNN variants. The best configuration is reported without multiplicity correction. While the consistent patterns observed—GATv2 outperforming GCN, centrality features consistently improving performance, *p* = 0.8 consistently optimal—suggest genuine effects rather than chance; future confirmatory studies should pre-register the optimal configuration and validate on independent data.

It is worth situating the SGNN approach within the broader landscape of methods that seek to capture complex inter-feature relationships in high-dimensional medical imaging data. Alternative paradigms include attention-based architectures such as transformers applied to tabular radiomic features, autoencoder-derived latent representations, and hybrid computational strategies that combine classical and non-classical learning frameworks to encode complex feature dependencies [46]. Each of these paradigms offers complementary strengths: transformers excel at global attention over feature sets, while graph-based methods such as SGNN encode pairwise relationships with explicit biological interpretability through the edge-weight construction mechanism. A systematic comparison of SGNN with these alternative approaches on the same radiomic dataset is a valuable direction for future work and would further clarify the conditions under which relational graph modelling provides a distinctive advantage over other interaction-aware architectures.

Additional limitations should be noted. First, PSA density, which was a significant baseline predictor in this cohort (*p* = 0.014; Table 1) and was included in the best-performing model of Sushentsev et al. [28], was included in the feature pool but its selection depended on the mutual information ranking within each fold. This data-driven approach may not have consistently prioritised PSAd, potentially limiting performance. Second, the critical limitation of this study is the absence of external validation. The model was developed and evaluated on a single-centre, single-scanner cohort (3T GE MR750, standardised protocol), which limits generalisability to settings with different acquisition parameters, patient selection criteria, or biopsy protocols. While the controlled acquisition environment reduces intra-cohort variability from scanner effects—a known confounder in radiomic studies—it does not provide evidence of performance in real-world multi-centre deployments. Future work should prioritise multi-centre external validation, with acquisition harmonisation using approaches such as ComBat [47] to control for scanner-related feature variability. Third, graph explainability analysis (e.g., GNNExplainer [44]) was not performed in this study and represents an important direction for future work, as it could identify which specific radiomic feature interactions are most predictive of progression and provide biologically interpretable insights. Fourth, while the Optuna budget of eight trials per configuration was constrained by computational resources, preliminary experiments indicated that performance plateaued within this range. Fifth, the evaluation protocol (5-fold CV) differs from the single train/test split used in other analyses of this cohort [28], which should be considered when comparing results across studies.

The retrospective binary classification design does not fully account for informative censoring: patients with shorter follow-up among the non-progressor group may include some who would have progressed with longer observation. A time-to-event analysis (Cox regression or survival-adjusted SGNN) would better characterise this uncertainty and is a worthwhile direction for future work.

While ICC-based feature selection (ICC > 0.8) with morphological perturbation addresses within-study segmentation variability, cross-institutional segmentation differences driven by variability in imaging resolution, reader training, and software are not captured by this approach and may affect the generalisability of both the radiomic features and the graph structures derived from them. Future work should assess SGNN robustness to inter-reader segmentation variability and evaluate automated segmentation pipelines as a means of standardising ROI delineation across institutions.

A natural extension of the SGNN framework is the incorporation of longitudinal radiomic data into dynamic graph structures. In the present study, each patient was represented by a single static graph constructed from baseline features. In a longitudinal extension, a sequence of patient-specific graphs—one per serial MRI time point—could be constructed, with edge weights encoding pairwise feature discriminability as it evolves over follow-up. Temporal graph neural networks combining graph message-passing with recurrent architectures (e.g., LSTM-GNN or GRU-GNN) could then learn how the inter-feature network structure changes over time, directly modelling the radiomic velocity of cancer progression. This architecture would be directly comparable to the delta-radiomic [29] and time-series LSTM [30] approaches that achieved AUCs of 0.91–0.92 on this cohort, while additionally encoding the relational structure of feature changes rather than treating each delta-radiomic feature in isolation. Further priorities include integration of additional clinical covariates (age, biopsy grade group, lesion zonal location) as graph-level features, application of graph explainability methods such as GNNExplainer [44] to identify the most prognostically important feature interactions, and incorporation of PSA trajectories into the graph construction framework.

## 5. Conclusions

The Synolitic Graph Neural Network framework achieved the highest mean ROC-AUC (0.699) among evaluated single-timepoint approaches for predicting prostate cancer progression on active surveillance. While this performance is numerically lower than that of dynamic longitudinal models previously reported for this cohort, it represents a meaningful proof-of-concept result in the inherently challenging baseline single-timepoint setting and warrants prospective validation. In this pre-selected low-risk population, where clinical and radiological indicators of progression have not yet fully manifested, the SGNN’s ability to outperform conventional machine learning by an absolute margin of 0.065 highlights the value of modelling inter-feature relationships. Graph centrality features and the GATv2 attention-based architecture consistently enhanced performance across configurations. The finding that end-to-end GNN learning is required to realise the benefit of graph-based modelling—whereas extracting graph summary statistics for conventional classifiers did not improve performance—highlights the importance of the full SGNN pipeline. These findings support graph-based modelling of radiomic inter-feature relationships as a complementary approach for quantitative image analysis in active surveillance, with future work prioritising longitudinal graph construction, graph explainability, and multi-centre validation.

## Figures and Tables

**Table 1:** Baseline clinicopathological characteristics of the study cohort. *p*-Values correspond to intergroup comparisons using the Mann–Whitney *U* test. IQR: interquartile range.

Parameter	Progressors (*n* = 73)	Non-Progressors (*n* = 270)	*p*-Value
PSA, ng/mL, median (IQR)	5.9 (4.5–8.1)	5.9 (4.2–7.8)	0.394
Gland volume, mL, median (IQR)	43.2 (35.0–54.5)	47.0 (36.1–67.2)	0.185
PSA density, median (IQR)	0.14 (0.10–0.21)	0.12 (0.08–0.16)	0.014
AS follow-up, months, median (IQR)	40 (27–56)	49 (36–74)	0.001

**Table 2:** Conventional ML performance (5-fold stratified cross-validation).

Model	ROC-AUC	F1-Score	Accuracy
Gradient Boosting	0.634 ± 0.080	0.229 ± 0.083	0.781 ± 0.033
Logistic Regression	0.625 ± 0.043	0.365 ± 0.052	0.630 ± 0.043
Random Forest	0.603 ± 0.030	0.090 ± 0.124	0.784 ± 0.023
SVM	0.593 ± 0.053	0.345 ± 0.051	0.577 ± 0.036

**Table 3:** SGNN performance across architectures, sparsification strategies, and node features (5-fold CV). Hyperparameters optimised via Optuna within training folds.

Architecture	Sparsification	Node Features	ROC-AUC
GATv2	sparsify_p (0.8)	Yes	0.699 ± 0.044
GATv2	sparsify_p (0.2)	Yes	0.692 ± 0.070
GCN	sparsify_p (0.8)	Yes	0.679 ± 0.067
GATv2	min_connected	Yes	0.668 ± 0.035
GCN	sparsify_p (0.2)	Yes	0.663 ± 0.031
GATv2	sparsify_p (0.8)	No	0.660 ± 0.069
GATv2	sparsify_p (0.2)	No	0.658 ± 0.056
GATv2	no_sparsify	Yes	0.649 ± 0.073
GCN	sparsify_p (0.8)	No	0.638 ± 0.069
GATv2	min_connected	No	0.638 ± 0.058
GCN	no_sparsify	Yes	0.636 ± 0.079
GCN	min_connected	Yes	0.629 ± 0.083

**Table 4:** Simplified SGNN performance with different feature counts (5-fold CV).

Features	Model	ROC-AUC
20	SGNN-GB	0.554 ± 0.096
20	SGNN-RF	0.534 ± 0.085
30	SGNN-GB	0.555 ± 0.092
30	SGNN-RF	0.538 ± 0.045
40	SGNN-GB	0.579 ± 0.079
40	SGNN-RF	0.548 ± 0.042

## Data Availability

The raw data supporting the conclusions of this article will be made available by the authors on request.
